# Heterochronic Developmental Shifts Underlying Squamate Cerebellar Diversity Unveil the Key Features of Amniote Cerebellogenesis

**DOI:** 10.3389/fcell.2020.593377

**Published:** 2020-10-22

**Authors:** Simone Macrì, Nicolas Di-Poï

**Affiliations:** Program in Developmental Biology, Institute of Biotechnology, University of Helsinki, Helsinki, Finland

**Keywords:** development, evolution, cerebellum, squamates, patterning

## Abstract

Despite a remarkable conservation of architecture and function, the cerebellum of vertebrates shows extensive variation in morphology, size, and foliation pattern. These features make this brain subdivision a powerful model to investigate the evolutionary developmental mechanisms underlying neuroanatomical complexity both within and between anamniote and amniote species. Here, we fill a major evolutionary gap by characterizing the developing cerebellum in two non-avian reptile species—bearded dragon lizard and African house snake—representative of extreme cerebellar morphologies and neuronal arrangement patterns found in squamates. Our data suggest that developmental strategies regarded as exclusive hallmark of birds and mammals, including transit amplification in an external granule layer (EGL) and Sonic hedgehog expression by underlying Purkinje cells (PCs), contribute to squamate cerebellogenesis independently from foliation pattern. Furthermore, direct comparison of our models suggests the key importance of spatiotemporal patterning and dynamic interaction between granule cells and PCs in defining cortical organization. Especially, the observed heterochronic shifts in early cerebellogenesis events, including upper rhombic lip progenitor activity and EGL maintenance, are strongly expected to affect the dynamics of molecular interaction between neuronal cell types in snakes. Altogether, these findings help clarifying some of the morphogenetic and molecular underpinnings of amniote cerebellar corticogenesis, but also suggest new potential molecular mechanisms underlying cerebellar complexity in squamates. Furthermore, squamate models analyzed here are revealed as key animal models to further understand mechanisms of brain organization.

## Introduction

The cerebellum is a prominent feature of the vertebrate hindbrain that varies extensively in terms of relative size and morphology not only across major vertebrate groups, but also in closely related species with distinct ecological and behavioral strategies (Voogd and Glickstein, 1998; Butler and Hodos, 2005; Striedter, 2005; Macrì et al., 2019). It reaches the highest level of morphological complexity in birds, mammals, and in some cartilaginous and bony fishes, in which a remarkable volume increase parallels a profound surface area expansion leading to a highly foliated structure (Butler and Hodos, 2005; Puzdrowski and Gruber, 2009; Sukhum et al., 2018). Remarkably, despite the observed variation in overall morphology, the basic features of cerebellar cytoarchitecture are relatively well-conserved across vertebrates (Larsell, 1967, 1970; Voogd and Glickstein, 1998; Butler and Hodos, 2005). Particularly, the cerebellar cortex is composed of a relatively small number of neuronal types, which are classified according to their function as excitatory or inhibitory neurons (Hashimoto and Hibi, 2012). The most important excitatory neurons include granule cells (GCs) and unipolar brush cells, and the inhibitory neurons include GABAergic Purkinje cells (PCs), Golgi cells, basket cells, and stellate cells. These neurons are arranged in a dense but well-defined trilaminar organization consisting of an inner granule layer (GL), middle PC layer (PCL), and outer molecular layer (ML) where PC dendrites receive GC axons. The orderly cellular layout and extensive connectivity of the cerebellum give rise to a massive signal processing capability that plays a crucial role in motor control and coordination but also in higher cognitive functions such as attention, memory, and language (Strick et al., 2009; Balsters et al., 2013; Buckner, 2013; Koziol et al., 2014; Baumann et al., 2015; Schmahmann, 2019). Owing to its relatively simple laminar organization, the cerebellum has been an attractive model for studying developmental patterns and functions of the central nervous system in multiple vertebrates, including basal lineages such as cyclostomes (Sugahara et al., 2016). Importantly, variations have been reported in the topographic arrangement of major cell layers, number of neurons, foliation pattern, and neuronal connectivity among vertebrate cerebella. Notably, the distinct spatial distribution of GCs in lampreys, sharks, and sturgeons as well as the scattered arrangement of PCs in some cartilaginous fishes, lungfishes, and snakes differs from the stereotyped organization found in teleosts, amphibians, archosaurs, and mammals (Butts et al., 2011; Yopak et al., 2017; Macrì et al., 2019). Furthermore, different cerebellar compartmental organization, as reflected by the presence/absence or heterogenous arrangement of discrete longitudinal stripes of PCs expressing similar markers such as aldolase C (also known as zebrin II; Voogd, 1967; Hawkes and Leclerc, 1987; Brochu et al., 1990; Aspden et al., 2015; Wylie et al., 2017; Yopak et al., 2017), but also by specific projections that give some individual peculiarities to these longitudinal compartments (Biswas et al., 2019; Na et al., 2019), have been observed in vertebrates. Finally, different developmental innovations in cellular behavior and signaling have been described among teleosts, chondrichthyans, and amniotes for generating transverse lobules and elaborate foliated cerebellar morphologies. As a result, although cerebellar development is well-known to rely on the spatiotemporal activity patterns of several key signaling pathways, the exact molecular and evolutionary mechanisms that govern the generation and arrangement of major neuronal types and foliation pattern are only partially resolved.

Developmental studies in birds and more recently in mammals have shown that the major divisions of the cerebellum, including the two cerebellar hemispheres and the medial region called vermis, originate from both the rhombomere 1 and the isthmus (also referred as rhombomere 0) and require gradients of fibroblast growth factor (FGF) signaling such as FGF8 for survival and differential development (Martínez et al., 2013; Watson et al., 2017). Furthermore, cerebellar neurons in birds and mammals originate from different germinal zones and migrate to their destination using radial and/or tangential migratory pathways. PC precursors (PCPs) and other GABAergic cells expressing specific markers such as the basic helix-loop-helix (bHLH) proneural gene *Ptf1* (Hoshino et al., 2005) stem from the ventricular zone (VZ) and follow a highly directed movement upon maturation, including radial migration toward the cerebellar pial surface for PCs. In contrast, GC precursors (GCPs) emerge from an atonal bHLH transcription factor 1 (*Atoh1*) expressing domain, the upper rhombic lip (URL; Alder et al., 1996; Wingate and Hatten, 1999; Wingate, 2001; MacHold and Fishell, 2005; Wang et al., 2005), and follow first a subpial tangential movement before producing post-mitotic GCs that migrate radially toward the VZ to form the internal GL (IGL). Although the two principal cerebellar germinal zones VZ and URL appear conserved at least within gnathostomes, the developmental pattern of neuronal precursors diverge among taxa (Hibi et al., 2017). Especially, GC development in mammals and birds includes a unique phase of *Atoh1*-mediated transit amplification in an external GL (EGL) during the initial tangential migration phase (Alder et al., 1996; Wingate and Hatten, 1999). Transit amplification is a widespread strategy in neural development that allows the fine-tuning of cell numbers, and such process has been linked to the evolution of highly foliated cerebella in vertebrates. However, the absence of a typical EGL in chondrichthyans and teleosts (Chaplin et al., 2010; Kani et al., 2010), two groups that include species with hugely foliated cerebella (Yopak et al., 2007; Sukhum et al., 2018), and the presence of a distinct, non-proliferative EGL in amphibians (Gona, 1972; Butts et al., 2014a) suggest that this transient structure is in fact a key developmental innovation found in birds and mammals. In addition to the different proliferative strategies, the presence/absence or even variations in the timing and/or complexity of migration patterns taken by post-mitotic neuronal derivatives likely reflect divergence in cerebellar morphology and spatial arrangement of cerebellar neurons within and between vertebrate groups (Hibi et al., 2017; Rahimi-Balaei et al., 2018; Macrì et al., 2019).

Among key signaling factors involved in early cerebellar development, analyses of mouse models have highlighted the fundamental role of *Atoh1* in determining GCP proliferation within the EGL in response to Sonic hedgehog (SHH) secreted from underlying PCs (Ben-Arie et al., 1997; Lewis et al., 2004). The importance of SHH pathway in cerebellar histogenesis has been further underscored by genetic analyses demonstrating that its level of activation controls both the size and foliation pattern of the cerebellum (Dahmane and Ruiz, 1999; Wallace, 1999; Wechsler-Reya and Scott, 1999; Corrales, 2004, 2006; Lewis et al., 2004). Morphogens belonging to the bone morphogenetic protein (BMP) family such as BMP4, BMP6, BMP7, and GDF7 have also been shown to regulate the proliferation, specification, and survival of cerebellar GCPs (Lee et al., 1998; Alder et al., 1999; Krizhanovsky and Ben-Arie, 2006; Su et al., 2006; Barneda-Zahonero et al., 2009; Tong and Kwan, 2013). Similarly, the pivotal role of retinoic acid receptor-related orphan receptor alpha (*Rora*) in the developing cerebellum has been thoroughly documented. Particularly, disruption of *Rora* in *staggerer* mutant mice (Sidman et al., 1962) leads to severe deficiencies in PC physiology, morphology, and survival (Sotelo and Changeux, 1974; Landis and Sidman, 1978; Herrup and Mullen, 1979), which negatively impact EGL persistence and proliferation and ultimately result in cerebellar hypoplasia (Sidman et al., 1962). In a reciprocal fashion, the EGL has been shown to be critical for the peculiar positioning of PCs in monolayer, indicating that cortical integrity relies on the correct development of URL generated cells (Miyata et al., 1997; Jensen et al., 2002; Jensen, 2004). Especially, many studies have evidenced the key role of the extracellular matrix molecule Reelin (RELN) produced by post-mitotic GCPs in PC spatial alignment, and complete deficiency of *Reln* gene or of components of the RELN signaling pathway such as RELN receptors [very-low-density-lipoprotein receptor (VLDLR) and apolipoprotein E receptor 2 (ApoER2)] or adapter protein disabled-1 (DAB1) causes similar defects in cerebellar architecture and function, including extensive PC disorganization and hypoplasia (Heckroth et al., 1989; D’Arcangelo et al., 1995; Sheldon et al., 1997; Ware et al., 1997; Gallagher et al., 1998; Trommsdorff et al., 1999). Importantly, although the relevance of these genes and cellular interactions are relatively well-conserved in mammals and birds, significant molecular differences exist in all anamniote species investigated so far, including chondrichthyans, non-teleost and teleost ray*-*finned fishes, as well as amphibians. Notably, the distinctions are primarily linked to the various strategies used to generate and amplify GCs, including the absence of proliferative *Atoh1*-positive EGL progenitors and/or *Shh*-dependent GCP expansion in the developing cerebellum of anamniotes (Chaplin et al., 2010; Kani et al., 2010), further suggesting that the transient, proliferative EGL is an amniote adaptation to increase cerebellar complexity. However, substantial gaps in the vertebrate phylogeny remain unexplored. Especially, the molecular underpinnings of early cerebellar development, including the status of the EGL and cortical layer interactions, are still unknown for non-avian reptiles such as squamates (lizards and snakes), which occupy a key phylogenetic position and represent a major portion of the amniote tree with over 10,000 species. Furthermore, although the general organization of the cerebellum, from local circuitry to broad connectivity, is highly conserved among mammals, birds, and reptiles (Nieuwenhuys, 1967; Nieuwenhuys et al., 1998), adult squamates exhibit a wide diversity of cerebellar shape, size, and neuronal pattern directly linked to their ecological behaviors (Larsell, 1926; Aspden et al., 2015; Macrì et al., 2019), thus representing an excellent model to understand the evolutionary origin, structure, function, development, and adaptation of the amniote cerebellum.

Here, we performed the first study assessing the morphological, cellular, and molecular characterization of the developing embryonic cerebellum in non-avian reptiles, using two “non-classical” model species—the bearded dragon lizard (*Pogona vitticeps*) and the African house snake (*Boaedon fuliginosus*)—representative of extreme cerebellar morphologies and neuronal arrangement patterns found in squamates (Macrì et al., 2019). Our analysis suggests that cellular and molecular mechanisms previously identified in the developing cerebellum of birds and mammals are likely well-conserved in all major amniote groups, including squamate reptiles. Particularly, our gene expression data indicate that the formation of a proliferating EGL is most probably a true amniote developmental adaptation, although independent from the cerebellar foliation pattern. Furthermore, direct comparison of our two models suggests the existence of variations from the common amniote developmental blueprint in terms of GC generation and PC patterning, thus enriching the multi-faceted strategies adopted in vertebrate cerebellar histogenesis. Especially, the observed heterochronic shifts in the timing and/or duration of URL activity and EGL maintenance in snakes is expected to alter the dynamics of molecular interaction between GCs and PCs. Most importantly, although further experimental demonstrations would be needed, our findings give new insights about the contribution of key signaling pathways, cellular spatiotemporal interactions, and histogenetic events in defining the number and arrangement of major cerebellar neurons in vertebrates. Altogether, this set of results indicate the importance of squamate models to further understand basic principles of brain organization and evolutionary mechanisms of cerebellar complexity, which, in turn, can inform us as to how brain evolution has enabled vertebrate ecological capability.

## Results

### Comparative Architecture of Cerebellum in *P. vitticeps* and *B. fuliginosus*

**FIGURE 1:**
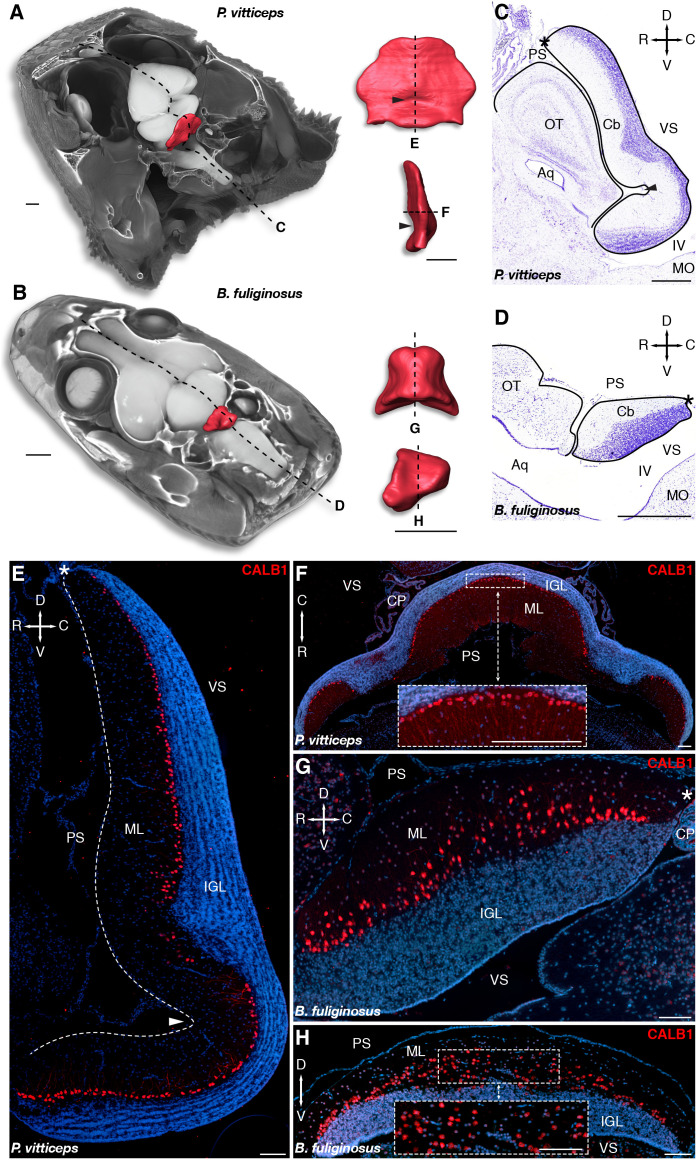
Morphology and cellular arrangement of bearded dragon lizard (*P. vitticeps*) and African house snake (*B. fuliginosus*) cerebellum. **(A,B)** 3D-volume rendering and high-resolution whole-brain segmentation of iodine-stained juvenile heads of *P. vitticeps* **(A)** and *B. fuliginosus* **(B)** highlighting the cerebellum structure (red color). High magnifications of 3D-rendered cerebella of *P. vitticeps* **(A)** and *B. fuliginosus* **(B)** are shown in pial (top) and lateral (bottom) views in the right column. Arrowheads indicate the position of the incomplete fissure in *P. vitticeps* cerebellum. Dashed lines and letters mark the sectioning planes relative to the histological preparations and immunostaining experiments in panels **(C,D)** and **(E–H)**, respectively. **(C,D)** Nissl staining of the cerebellum and neighboring brain regions in *P. vitticeps* **(C)** and *B. fuliginosus* **(D)**. Black lines demarcate the contour of the cerebellum and adjacent brain regions. The arrowhead in panel **(C)** indicates the position of the incomplete fissure in *P. vitticeps* cerebellum, and asterisks in panels **(C,D)** indicate the position of the embryonic upper rhombic lip. Crossed arrows point toward rostral (R), caudal (C), dorsal (D), and ventral (V) directions. **(E–H)** Immunodetection of Purkinje cells (PCs) with CALB1 marker, using sagittal **(E)** or axial **(F)** sections of *P. vitticeps* and sagittal **(G)** or coronal **(H)** sections of *B. fuliginosus* juvenile cerebellum (red staining). Cell nuclei are counterstained with DAPI (blue staining). The arrowhead in panel **(E)** indicates the position of the incomplete fissure, and the white dashed line delimitates the cerebellar pial surface. Asterisks in panels **(E,G)** indicate the position of the embryonic upper rhombic lip. Insets in panels **(F,H)** show high magnifications of PC spatial organization. Crossed white arrows point toward rostral (R), caudal (C), dorsal (D), and ventral (V) directions. OT, optic tectum; Cb, cerebellum; Aq, aqueduct; MO, medulla oblongata; IV, fourth ventricle; PS, pial surface; ML, molecular layer; IGL, internal granule layer; VS, ventricular surface; CP, choroid plexus. Scale bars: 1 mm **(A,B)**, 500 μm **(C,D)**, 100 μm **(E–H)**.

Despite its high levels of functional conservation, the cerebellum displays a wide range of morphological variation across vertebrates (Larsell, 1923, 1926, 1932; Voogd and Glickstein, 1998; Butler and Hodos, 2005; Striedter, 2005). Among amniotes, mammals and birds exhibit highly convoluted cerebellar architectures, whereas non-avian reptiles such as lizards and snakes feature less elaborated, unfoliated structures (Larsell, 1926; Nieuwenhuys, 1967; Nieuwenhuys et al., 1998). Exceptions include crocodilians and a few lizard species, in which one or two transverse fissures that divide the cerebellum into different lobes were observed (Larsell, 1926, 1932). Past and recent studies (Larsell, 1926; Macrì et al., 2019), however, revealed an extraordinarily rich gamut of cerebellar morphologies and cortical organization in squamates paralleling their exceptional ecomorphological diversification, thus highlighting the potential of this model for elucidating key processes underlying vertebrate brain evolution and development. To compare the distinctive morphological characteristics of the cerebellum in two squamate species with different ecological behaviors—one lizard (*P. vitticeps*) and one snake (*B. fuliginosus*), we used high-definition 3D reconstructions of whole-brains and isolated cerebella based on contrast-enhanced computed tomography (CT; Figures 1A,B) as well as histological stainings of brain sections (Figures 1C,D). A substantial divergence of cerebellar shape, size, and spatial relationships with other brain subdivisions is evident between the two model species. Especially, besides the three-fold reduction in cerebellum volume observed in *B. fuliginosus* when compared to *P. vitticeps*, the snake exhibits a relatively small trapezoidal cerebellum, in contrast to the lizard leaf-shaped structure (Figures 1A–D). Furthermore, the presence of an incomplete fissure on the pial surface in *P. vitticeps* imparts a marked inversion in cerebellar tilting relative to the brain anatomical planes (Figures 1A,C,E). Consequently, *B. fuliginosus* cerebellum lies almost completely embedded in the 4th ventricle, whereas the lizard counterpart is dorsally elongated and bends over the tectal hemispheres, toward the rostral edge of the brain (Figures 1A–D). At the cellular level, our characterization of major cerebellar neuron types such as PCs and GCs confirms alternative spatial arrangement of PCs in the two species (Macrì et al., 2019), as revealed here by immunodetection of PC marker such as calbindin 1 (CALB1; Figures 1E–H). The lizard cortex shows a well-defined trilaminar organization, including a clearly distinguishable PC layer composed of cells neatly distributed along the outer border of the IGL (Figures 1E,F), as described for birds and mammals. In contrast, *B. fuliginosus* PCs appear scattered due to their arrangement in radially oriented columns, each containing a varying number of cells and protruding with different extent in the ML (Figures 1G,H). Altogether, these observations corroborate previous qualitative and quantitative descriptions of squamate cerebella, including heterogeneity in morphological features and PC spatial layouts among lizard and snake species (Larsell, 1926; Aspden et al., 2015; Wylie et al., 2017; Hoops et al., 2018; Macrì et al., 2019), and confirm the unique neuroanatomical landscape of this structure in squamates.

### Generation and Patterning of Cerebellar PCs in *P. vitticeps*

**FIGURE 2:**
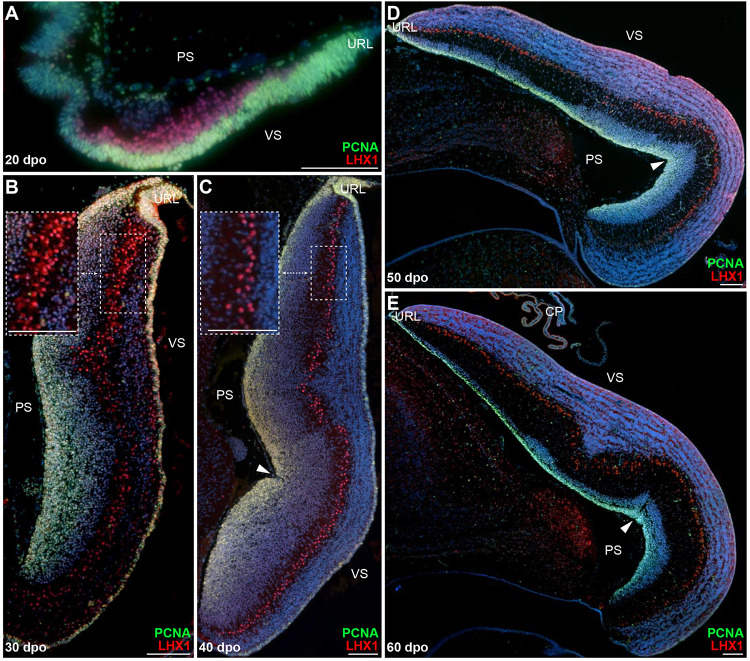
Proliferation pattern and PC development in embryonic *P. vitticeps* cerebellum. **(A–E)** Double immunohistochemistry (IHC) staining for PCNA (green staining) and LHX1 (red) markers at various developmental stages, indicated as embryonic days post-oviposition (dpo), in the cerebellum of *P. vitticeps*. Arrowheads in panels **(C–E)** indicate the position of the incomplete fissure on the cerebellar pial surface. Insets in panels **(B,C)** show high magnifications of PC spatial organization. PS, pial surface; VS, ventricular surface; URL, upper rhombic lip. Scale bars: 100 μm.

The distinct neuron types of vertebrate cerebellum have well-defined, conserved developmental origins, including the URL and VZ germinal zones, and migrate to their destination using radial and/or tangential migratory pathways (Butts et al., 2011). The initial GABAergic progenitors giving rise to PCs emerge from the proliferative VZ epithelium (Altman and Bayer, 1997; Sotelo, 2004; Hoshino et al., 2005), and migrate radially toward the cerebellar pial surface as they mature, eventually acquiring both their physiological and morphological features as well as their monolayer arrangement typical of most amniote cerebella. To investigate PC developmental program in squamates, we first analyzed the lizard model, which shows a canonical spatial alignment of PCs at postnatal stages (Figures 1E,F). Immunolabelings of developing cerebella were performed at various post-ovipositional embryonic stages, using the PC lineage marker LIM homeobox protein 1 (LHX1; Zhao et al., 2007) in combination with proliferating cell nuclear antigen (PCNA) to get insights on the proliferative potential of the VZ epithelium. At 20 days post-oviposition (dpo), the earliest stage at which the developing cerebellar primordium can be clearly distinguished in our lizard model, LHX1-positive post-mitotic PCPs exit a highly proliferative VZ and migrate in a radially oriented fashion toward the outer pial surface (Figure 2A). Ten embryonic days later (30 dpo; Figure 2B), the VZ is still actively proliferating, and immature PCs aggregate as multi-layered sigmoidal PC clusters (PCCs) lying in an intermediate position along the ventricular-pial axis of the cerebellum. At this stage, the cerebellum appears elongated and thickened, and a second proliferative, pluristratified domain lines the entire length of pial surface in continuation with the URL (Figure 2B). At 40 dpo, proliferation attenuates on the ventricular surface, while both the URL and pial surface are still sites of sustained PCNA labeling (Figure 2C). PCs are more evenly spaced than in previous stages, and PCCs start dispersing to acquire a less stratified appearance (compare insets in Figures 2B,C). Furthermore, as noticed in juvenile lizards (Figures 1C,E), the 40 dpo stage features the formation of an incomplete and shallow fissure on the medial pial surface. As development proceeds, PCs continue their alignment process (Figure 2D), and by the time of hatching (60 dpo), they reach an almost continuous monolayer configuration (Figure 2E). Although the ventricular epithelium has exhausted its proliferative potential at 60 dpo, proliferation is still active on the pial cerebellar surface (Figure 2E), and a PCNA-positive domain of progressively reducing thickness is detected at least until two weeks post-hatching (Figure 2E and Supplementary Figure 1A). Together, these results indicate that PC patterning in our lizard model largely occurs at embryonic stages according to a similar ground plan than described in other amniotes.

### Developmental Patterning of Cerebellar GCs and EGL Formation in *P. vitticeps*

**FIGURE 3:**
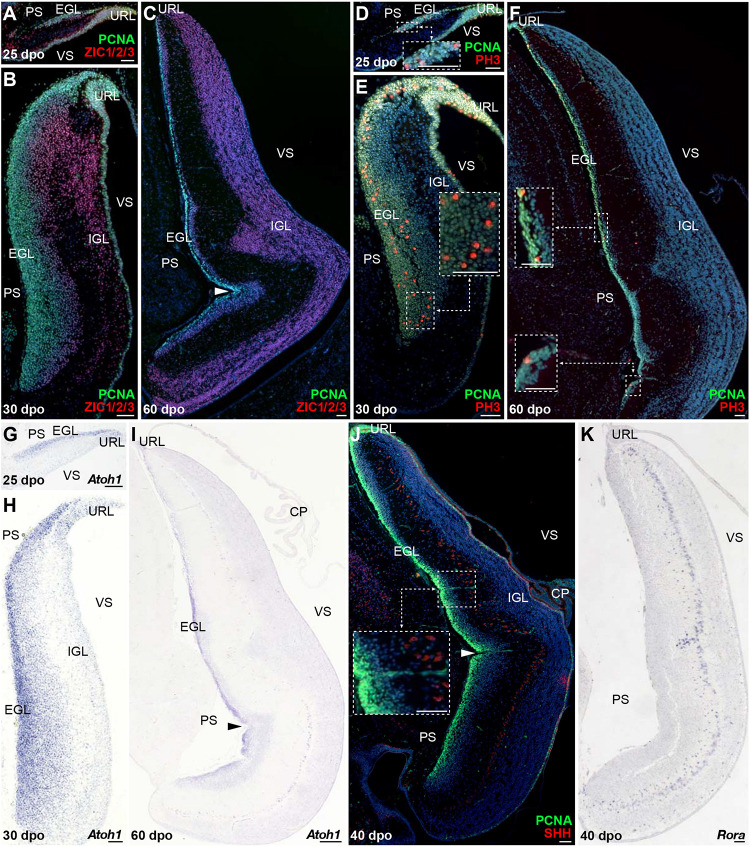
Molecular characterization of GC patterning in *P. vitticeps*. **(A–F)** Double IHC for PCNA (green staining) with ZIC1/2/3 [**(A–C)**; red] or PH3 [**(D–F)**; red] markers at various indicated embryonic developmental stages (25, 30, and 60 dpo) in the cerebellum of *P. vitticeps*. Insets in panels **(D–F)** show high magnifications of mitotic progenitors on the pial surface. **(G–I)** *In situ* hybridization (ISH) showing the expression of *Atoh1* at various indicated embryonic developmental stages (25, 30, and 60 dpo). **(J,K)** Double IHC for PCNA (green) and SHH [**(J)**; red] or ISH for *Rora* **(K)** at 40 dpo. The inset in panel **(J)** shows high magnification of SHH-positive PCs. Arrowheads in **(C,I,J)** indicate the position of the incomplete fissure on the cerebellar pial surface. PS, pial surface; EGL, external granule layer; IGL, internal granule layer; VS, ventricular surface; URL, upper rhombic lip; CP, choroid plexus. Scale bars: 50 μm.

Cerebellar GCs, the most abundant neurons of the vertebrate brain, differentiate from precursors generated in a highly germinative region—the URL, situated at the junction between the posterior rim of the developing cerebellar primordium and the roof plate (RP) of the fourth ventricle (Hallonet and Le Douarin, 1993; Ryder and Cepko, 1994; Alder et al., 1996; Hatten et al., 1997; Wingate, 2001). In amniotes, GCPs produced by the URL follow an elaborated migratory program, before settling in the IGL as terminally differentiated GCs. In their first, tangential translocation phase, GCPs migrate along the entire extent of the cerebellar pial surface to form a pluristratified domain—the EGL, before undertaking a second, radially oriented, migratory step along the pial-to-ventricular cerebellar axis. The EGL is a highly proliferative, transient germinal zone responsible for the exponential amplification of GCPs, but its formation has only been documented in birds and mammals so far (Gona, 1972; Alder et al., 1996; Wingate and Hatten, 1999; Chaplin et al., 2010). Consequently, whether a proliferative EGL is an exclusive hallmark of some vertebrate groups or a feature shared by all amniotes remains unknown. To clarify this issue, we explored the patterns of GC generation in our lizard model, with a particular focus on the EGL-like features of the highly proliferating layer detected on cerebellar pial surface. We first immunostained *P. vitticeps* embryonic cerebella at representative stages (see Figure 2), using markers of GC lineage such as Zic family member 1/2/3 (ZIC1/2/3; Aruga et al., 1994, 1998) in combination with PCNA. From 20 to 30 dpo, a rapid expansion of PCNA-positive cells occurs on the pial surface of developing cerebellum, with ZIC1/2/3-positive post-mitotic GCPs starting to delaminate and migrate radially out from this zone as early as 25 dpo (Figures 3A,B). As development proceeds, GCs steadily accumulate on the opposite (ventricular) side of the cerebellum, resulting in the formation of a more delineated IGL, and the two proliferating URL and pial domains gradually thin out (Figures 3B,C). By the time of hatching (60 dpo), although a reduced proliferative activity remains on pial surface until early postnatal stages (Supplementary Figure 1A), the IGL already consists of a multilayered arrangement of tightly packed GCs, and its thickness has dramatically increased (Figure 3C). Importantly, these data clearly connotate the transient proliferative domain on pial surface as a genuine EGL, thus suggesting striking analogies in GC developmental strategies between squamates, birds, and mammals. Furthermore, the observed persistence of proliferation beyond hatching stage in *P. vitticeps* indicate finalization of cerebellar architecture at early postnatal life, thus resembling mouse cerebellogenesis.

To further confirm the presence of a proliferative EGL in squamates, we assessed the expression pattern of additional proliferative and molecular markers well-known to play a critical role in both EGL formation and maintenance in birds and mammals. Especially, the EGL is defined not only by its active, transient mitotic activity, but also by the expression of *Atoh1* (Akazawa et al., 1995; Ben-Arie et al., 1996, 1997) that is absolutely required both for GCP amplification and identity (Flora et al., 2009; Klisch et al., 2011). Furthermore, EGL precursors require SHH supplied by the underlying PCs to expand the GC population and achieve the correct cerebellar size and extent of foliation (Dahmane and Ruiz, 1999; Wallace, 1999; Wechsler-Reya and Scott, 1999; Corrales, 2004, 2006; Lewis et al., 2004). As revealed by co-immunodetection of PCNA with the phosphorylated form of histone H3 (PH3), a specific marker for cells undergoing mitosis, the lizard EGL displays PCNA/PH3 double-positive cells indicative of mitotic events both in regions close to the URL as well as along the subpial stream of GC precursors (Figures 3D–F). In particular, at 25 dpo mitotic cells are found at different levels along the rostro-caudal EGL extent, including its rostral edge (Figure 3D). As cerebellum develops, cell divisions become more prominent and, like recently observed in chicken (Hanzel et al., 2019), also occur in more internal EGL regions (Figure 3E). Moreover, actively dividing cells are still detected even far from the URL region by 60 dpo (Figure 3F). Similarly to the situation in other amniotes, *in situ* hybridization (ISH) staining against *Atoh1* strongly labels *P. vitticeps* URL and EGL at all embryonic stages investigated, with an expression profile that precisely overlaps the spatiotemporal evolution of these two domains (Figures 3G–I). Furthermore, SHH protein expression is detected in PCs starting from 40 dpo, a stage where the EGL contains three to five layers of cells (Figure 3J). To corroborate SHH detection timing, we analyzed by ISH the expression pattern of the transcription factor *Rora*, one direct *Shh* modulator involved PC maturation, survival, and lifelong morpho-functional integrity (Sidman et al., 1962; Hamilton et al., 1996; Dussault et al., 1998; Gold et al., 2003, 2007; Chen et al., 2013; Takeo et al., 2015). As anticipated, *Rora* expression pattern perfectly matches SHH immunostaining in PCs, being detected from 40 dpo till adulthood in *P. vitticeps* (Figure 3K and Supplementary Figure 1B), thus confirming SHH spatiotemporal pattern. Altogether, our results strongly suggest the formation of a proliferative EGL structure on the pial surface of lizard cerebellum, likely yielding the generation of a vast number of GCs through a secondary transit-amplifying phase, thus indicating that squamates feature developmental milestones thought to be exclusive of birds and mammals.

### Patterning and Generation of Cerebellar Neurons in *B. fuliginosus*

**FIGURE 4:**
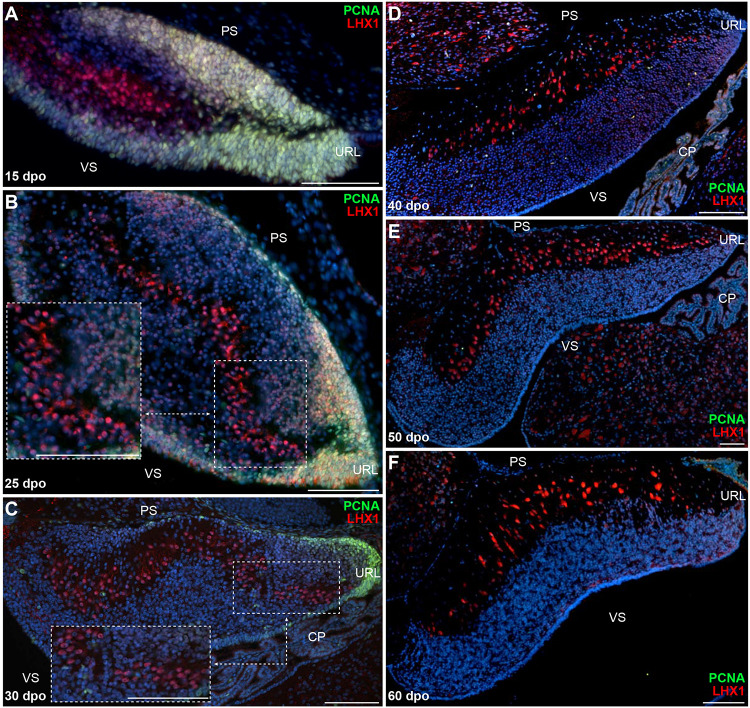
Proliferation pattern and PC development in embryonic *B. fuliginosus* cerebellum. **(A–F)** Double IHC for PCNA (green staining) and LHX1 (red) markers at various developmental embryonic stages between 15 and 60 dpo in the cerebellum of *B. fuliginosus*. Insets in panels **(B,C)** show high magnifications of PC spatial organization. PS, pial surface; VS, ventricular surface; URL, upper rhombic lip; CP, choroid plexus. Scale bars: 100 μm.

Owing to the divergent architecture and size of postnatal cerebellum in our two squamate models (see Figure 1), we then explored the cellular and molecular dynamics of *B. fuliginosus* neuronal development to identify possible mechanisms that could explain their peculiar organization. At early embryonic stages, although a cerebellar primordium showing a highly proliferative VZ can be distinguished at 12 dpo, PCNA-positive cells are only found on both cerebellar surfaces at 15 dpo, a stage when groups of LHX1-positive PCs have started to migrate out from the ventricular epithelium (Figure 4A). It is worth noting that the overall cerebellogenesis was anticipated to initiate earlier in *B. fuliginosus* than in *P. vitticeps* because of the relative advanced development of snake embryos at oviposition (Boback et al., 2012; Ollonen et al., 2018). At 25 dpo, PCs aggregate to form pluristratified PCCs in the middle of the developing cerebellum along the ventricular-pial axis, similarly to the situation observed in *P. vitticeps* at mid-embryonic stage, and the ventricular surface display less proliferative activity (Figure 4B). As cerebellum morphogenesis progresses, clear differences emerge in the snake PC developmental program when compared to the lizard counterpart. *B. fuliginosus* PCs fail to disperse and to undergo the complex spatial rearrangement observed during the last third of post-ovipositional development in *P. vitticeps*, but rather maintain a pluristratified, scattered configuration throughout embryogenesis (Figures 4C–F), a phenotype coherent with the observed juvenile situation (Figures 1G,H). The slight modifications observed in PC layout after 30 dpo (Figures 4D–F) are likely to be ascribed to consolidation of cerebellar neural wiring rather than to PC-autonomous dynamics. Compared to *P. vitticeps*, remarkable differences are also evident in the proliferative activity occurring on the pial surface. Especially, the number of PCNA-positive cells on the rostral half of this cerebellar side has already strongly reduced by 25 dpo, becoming almost entirely confined to the caudal third and URL of the cerebellum at 30 dpo (Figure 4C), and no proliferating cells are detected by 40 dpo (Figures 4D–F). This comparative analysis of neuron patterning indicates that, while the initial steps of PC radial migration and PCC formation appear conserved in our two squamate models, the alternative cortical organization in snakes is already determined during embryogenesis by a different PC capability to uniformly disperse from the multilayered cluster configuration.

**FIGURE 5:**
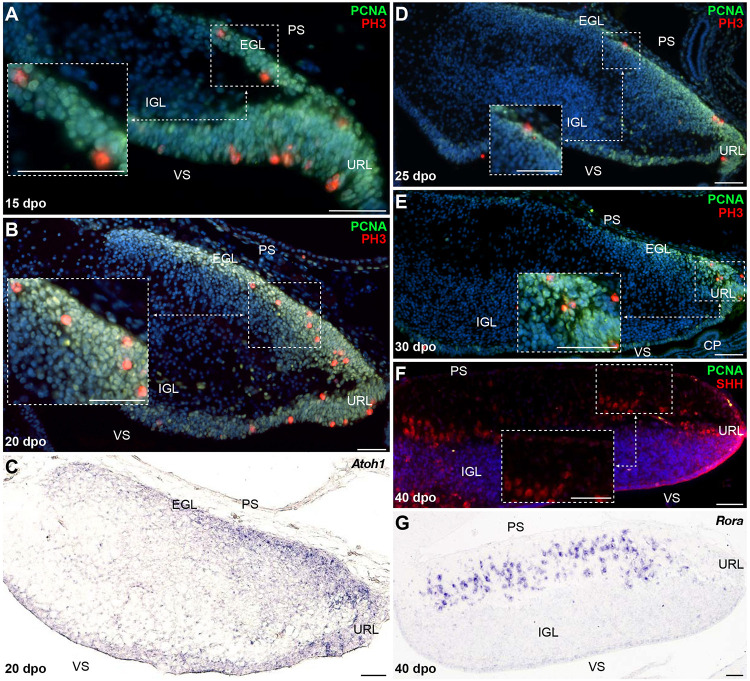
Molecular characterization of GC patterning in *B. fuliginosus*. **(A–E)** Double IHC for PCNA (green staining) and PH3 (red) markers **(A,B,D,E)** or ISH for *Atoh1* **(C)** at various indicated embryonic developmental stages between 15 and 30 dpo in the cerebellum of *B. fuliginosus*. Insets in panels **(A,B,D,E)** show high magnifications of mitotic progenitors on the pial surface. **(F,G)** Double IHC for PCNA (green) and SHH [**(F)**; red] or ISH for *Rora* **(G)** at 40 dpo. The inset in panel **(F)** shows high magnification of SHH-positive PCs. PS, pial surface; EGL, external granule layer; IGL, internal granule layer; VS, ventricular surface; URL, upper rhombic lip; CP, choroid plexus. Scale bars: 50 μm.

When compared to *P. vitticeps*, the observed changes in the timing of PCNA labeling along the snake pial surface suggest variations in embryonic cerebellogenesis and EGL maintenance among squamate species. To confirm this hypothesis, we next assessed the status of GC patterning and EGL formation in snakes, using proliferative and molecular markers described above for lizards. Our immunohistochemistry (IHC) and ISH experiments suggest that the same molecular framework underlies lizard and snake GC generation but, as expected, remarkable differences in the timing of major cerebellogenesis events differentiate the two models. As observed in *P. vitticeps*, a multilayered *Atoh1*-positive, proliferative EGL, featuring mitotic cells both at its rostralmost edge and in more internal layers, is found in the snake starting from 15 dpo (Figures 5A,B). As cerebellogenesis continues, however, a progressive reduction in both EGL thickness and rostrocaudal extent is accompanied by a decrease in the number of mitotic cells that becomes gradually confined to the caudal edge immediately adjacent to the URL (20-30 dpo; Figures 5C–E), and no proliferative activity is detected on the pial surface as well as in the URL by 40 dpo (Figure 5F). The EGL germinative potential in the snake, thus, significantly differs from the proliferative pattern of *P. vitticeps* that persists for a much longer embryonic period and even beyond (Supplementary Figure 1A). Surprisingly, however, the 40 dpo stage features the initial detection of both SHH and *Rora* in snake PCs, regardless of EGL complete disappearance, indicating that similar molecular events than in *P. vitticeps* occur during PC maturation in snakes (Figures 5F,G and Supplementary Figure 1C). Furthermore, despite the initial shift in embryonic timing of early neuron patterning in terms of dpo, these observations suggest that cerebellum development is synchronized in terms of PC maturation stage at 40 dpo. Altogether, these findings further indicate that cellular and molecular mechanisms such as EGL formation and SHH expression by PCs are not an exclusive trait of birds and mammals but also a prominent feature of squamate cerebellogenesis. Interestingly, however, the absence of EGL at the onset of SHH expression indicates the absence of secondary, PC-induced transit-amplifying phase in snakes, a phenotype already observed in mutant mice with complete abrogation of SHH signaling (Corrales, 2006) and coherent with the rapid decline of EGL structure.

### Molecular Mechanisms Regulating Initial Formation and Maintenance of EGL in Squamates

**FIGURE 6:**
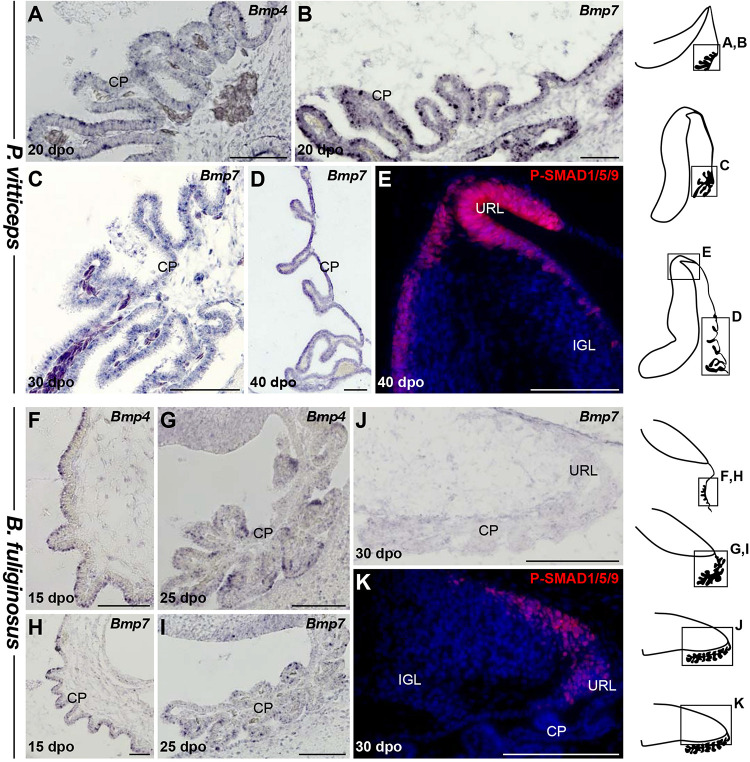
Characterization of BMP signaling pathway during early cerebellogenesis in *P. vitticeps* and *B. fuliginosus*. **(A–D)** ISH showing the expression of *Bmp4* **(A)** or *Bmp7* **(B–D)** at various indicated embryonic developmental stages in non-neural tissues bordering the cerebellum of *P. vitticeps*. **(E)** IHC for P-SMAD1/5/9 in the dorsal part and URL region of *P. vitticeps* cerebellum at 40 dpo. **(F–I)** ISH showing the expression of *Bmp4* **(F–J)** or *Bmp7* **(H–J)** at various indicated embryonic developmental stages in non-neural tissues bordering the cerebellum of *B. fuliginosus*. **(K)** IHC for P-SMAD1/5/9 (red staining) in the caudal part and URL region of *B. fuliginosus* cerebellum at 30 dpo. IGL, internal granule layer; URL, upper rhombic lip; CP, choroid plexus. Scale bars: 100 μm.

We next aimed at assessing the molecular mechanisms that could explain the observed changes in URL proliferation activity and EGL formation between our two squamate species. Early cerebellar development relies on signaling molecules secreted by bordering non-neural tissues, including the choroid plexus (CP) and RP of the fourth ventricle (Yamamoto et al., 1996; Liu and Joyner, 2001; Wurst et al., 2001; Chizhikov et al., 2006; Krizhanovsky and Ben-Arie, 2006). In the context of GC development, BMP signaling from these non-neural areas plays a critical role in URL induction and generation of GCPs that migrate toward the EGL (Chizhikov et al., 2006; Krizhanovsky and Ben-Arie, 2006; Qin et al., 2006; Tong et al., 2015), and the potential of BMP ligands such as BMP7, GDF7, and BMP4 to regulate GCP proliferation and specification has been thoroughly documented both in cell/tissue culture assays and *in vivo* (Alder et al., 1996, 1999; Lee et al., 1998; Krizhanovsky and Ben-Arie, 2006; Su et al., 2006; Salero and Hatten, 2007). Additionally, activation of BMP canonical signaling through SMAD proteins has been reported in embryonic URL and EGL (Fernandes et al., 2012; Owa et al., 2018), and SMAD1/5 double-mutant mice display defects in URL development as well as a reduced EGL accompanied by PC spatial disorganization (Tong and Kwan, 2013). In light of these data, we tested whether the altered proliferative patterns in both URL and EGL of our snake model could derive from modifications in the temporal and/or spatial expression pattern of BMP ligands (Figures 6A–K). Strikingly, while *Bmp4* and *Bmp7* transcripts are initially found concomitantly with URL activation in the CP of both species (Figures 6A,B,F,H), our ISH experiments indicate a precocious downregulation of these genes in the latter region during *B. fuliginosus* cerebellogenesis. In contrast to *P. vitticeps* that still maintains expression of *Bmp* genes by 40 dpo (Figures 6C,D), only a barely detectable level is noticed beyond initial PCC formation in snakes (Figures 6G-J). Importantly, these differences in *Bmp* expression parallel variations in phosphorylated forms of SMAD1/5/9, reflecting activity of canonical BMP signaling, which are only faintly detected and rapidly restricted to the shrinking EGL and URL domains in snakes when compared to the broad, abundant expression pattern in lizards (Figures 6E,K). These data suggest a tight link between BMP secretion by extra-cerebellar, non-neural tissues and both URL and EGL spatiotemporal dynamics in squamates. While the overall gene expression and activation domains in *P. vitticeps* resemble the situation described in other amniotes, a heterochronic shift in the timing and/or duration of URL activity is likely linked to the disappearance of EGL structure before PC maturation in snakes, eventually leading to absence of PC-induced transit-amplifying phase and EGL maintenance at subsequent developmental stages.

### Molecular Mechanisms Regulating Cerebellar Cortex Lamination in *P. vitticeps* and *B. fuliginosus*

**FIGURE 7:**
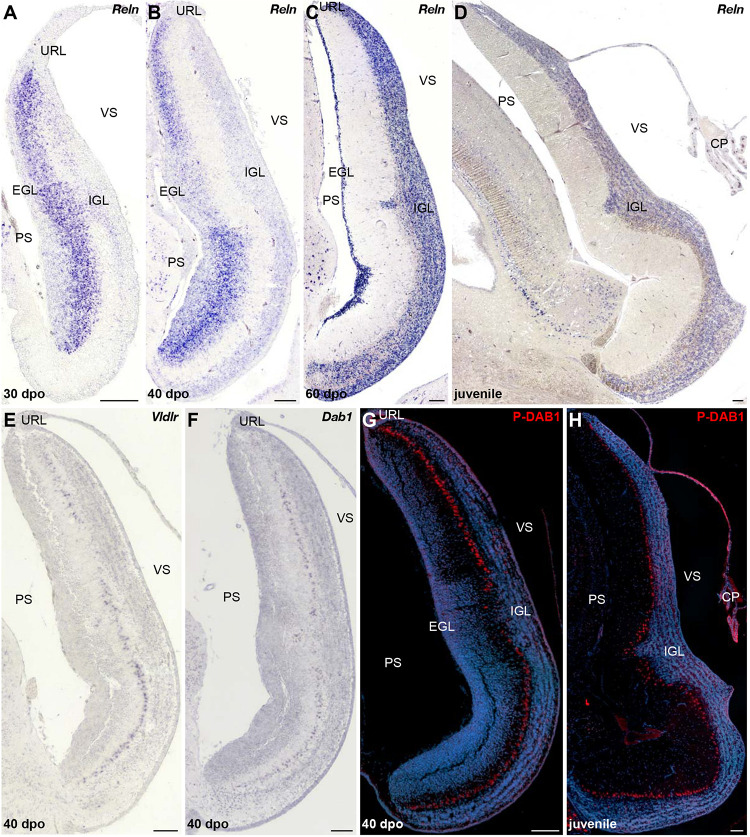
Characterization of RELN signaling pathway in the developing cerebellum of *P. vitticeps*. **(A–D)** ISH for *Reln* at various indicated embryonic (30, 40, and 60 dpo) and juvenile stages in the cerebellum of *P. vitticeps*. **(E,F)** ISH for *Vldlr* **(E)** or *Dab1* **(F)** at 40 dpo. **(G,H)** IHC for P-DAB1 (red staining) at 40 dpo **(G)** and juvenile **(H)** stages. PS, pial surface; EGL, external granule layer; IGL, internal granule layer; VS, ventricular surface; URL, upper rhombic lip; CP, choroid plexus. Scale bars: 100 μm.

**FIGURE 8:**
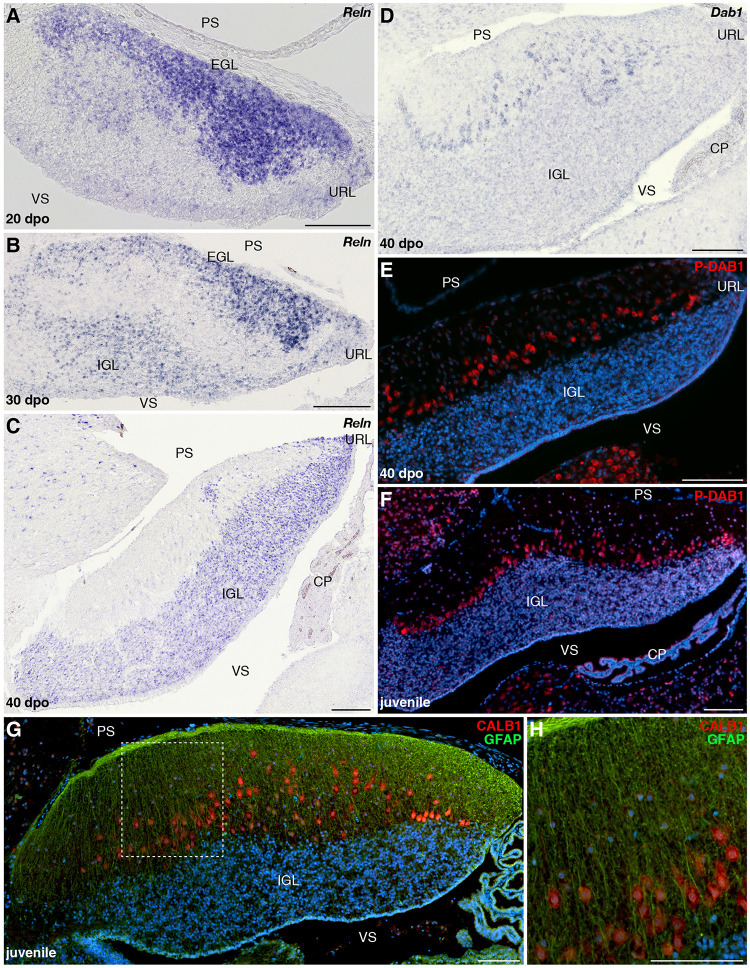
Developmental characterization of RELN signaling pathway and mature glial fiber organization in the cerebellum of *B. fuliginosus*. **(A–D)** ISH for *Reln* **(A–C)** or *Dab1* **(D)** at various indicated embryonic stages (20, 30, and/or 40 dpo) in the cerebellum of *B. fuliginosus*. **(E,F)** IHC for P-DAB1 (red staining) at 40 dpo **(E)** and juvenile **(F)** stages. **(G,H)** Double IHC for CALB1 (red) and GFAP (green) in the cerebellum of juvenile *B. fuliginosus*. A high magnification view of the area contained in the dashed rectangle in panel **(G)** is shown in panel **(H)**. PS, pial surface; EGL, external granule layer; IGL, internal granule layer; VS, ventricular surface; URL, upper rhombic lip; CP, choroid plexus. Scale bars: 100 μm.

Although PCs and GCs arise from separate progenitor zones, complex interactions between these two cell types play pivotal roles during cerebellar morphogenesis. Therefore, our results suggest that the divergent EGL developmental states featured by our models could be, at least in part, associated with the final differential distribution of PCs. The acquisition of the stereotypic monolayer configuration by bird and mammalian PCs relies on a well-characterized molecular cascade triggered by the molecule RELN, a large glycoprotein secreted by radially migrating post-mitotic GCPs in the developing ML as well as by terminally differentiated GCs already settled in the IGL (Caviness and Rakic, 1978; D’Arcangelo et al., 1995; Miyata et al., 1997; Pesold et al., 1998; Jensen et al., 2002). Based on genetic and biochemical studies, two RELN receptors have been identified, including VLDLR that is particularly highly expressed in cerebellar PCs (D’Arcangelo, 2014). Upon RELN binding, these receptors mediate the phosphorylation of the adaptor protein DAB1 that drives cytoskeletal rearrangements in PCs, eventually leading to the peculiar amniote PC pattern along the outer IGL border. Impairment at any level of this pathway leads to similar defects in cerebellar architecture, including severe alteration of PC topographic organization, and major locomotor deficits (D’Arcangelo et al., 1995; Sheldon et al., 1997; Ware et al., 1997; Gallagher et al., 1998; Trommsdorff et al., 1999). We then combined IHC and ISH experiments to identify potential variations in RELN signaling network that could determine the alternative cortical PC layout featured by our models. In *P. vitticeps*, *Reln* transcripts are initially particularly abundant in sparse post-mitotic GCPs delaminating from the EGL and then in a larger number of radially migrating post-mitotic GCPs (30 dpo), reflecting the massive progenitor expansion within EGL at early stages of cerebellum development (Figure 7A and data not shown). At 30 dpo, a faint staining is also detected on the ventricular side where early generated GCs start to colonize the presumptive IGL. As cerebellogenesis progresses, the *Reln* pattern strictly follows both EGL shrinking and continuous accumulation of GCs in the IGL (Figures 7B,C). Like in mammals, *Reln* expression persists in the IGL of juvenile lizards (Figure 7D), where it likely promotes synaptic plasticity and modulates neurotransmitter release in a similar fashion of neuronal subsets residing in adult neocortex and hippocampus (Beffert et al., 2005; Herz and Chen, 2006; Hellwig et al., 2011). However, in contrast to the expression of components of the RELN pathway that initiates at early stage of EGL formation in rodent models (Rice et al., 1998; Trommsdorff et al., 1999), the expression pattern of *Vldlr* and *Dab1* appears relatively delayed in *P. vitticeps*, being only detected in migrating PCs beyond initial lizard PCC formation, from 40 dpo onward (Figures 7E,F). Coherent with that, the phosphorylated form of DAB1 (P-DAB1), a molecular read-out of RELN signaling activity, perfectly matches *Vldlr* and *Dab1* expression in lizards (Figures 7G,H). Interestingly, the gene expression profile of RELN pathway in *B. fuliginosus* matches the asynchronous pattern featured by the lizard, with strong *Reln* labeling being detected in GCPs delaminating from EGL at early developmental stages as well as in GCs already settled in the IGL from later stages till adulthood (Figures 8A–C and Supplementary Figure 1D). Furthermore, both *Dab1* mRNA and P-DAB1 are detected starting from 40 dpo in snake PCs, confirming the synchronized expression profile of squamate PCs at this stage, and their expression is further maintained at postnatal stages (Figures 8D–F).

Despite the observed activation of DAB1 at similar embryonic stage in both squamate models, the onset of PC response to RELN signals occurs in a radically divergent morphogenetic context in the two species. At 40 dpo, GCP generation from URL and EGL is still sustained in *P. vitticeps*, and the relatively high level of *Reln* expression observed in delaminating and migrating GCPs likely provides a permissive environment for PC spatial rearrangement (Figure 7B). On the opposite cerebellar ventricular surface, the forming lizard IGL rather shows a reduced level of *Reln* at 40 dpo, indicating that RELN-responding PCs at this stage are exposed to a gradient of gene expression with its highest level on the pial side. At subsequent stages, *Reln* expression becomes progressively exclusive to the IGL, in correlation with EGL shrinking and ML thickening (Figures 7C,D). In contrast, the snake EGL already disappeared from developing cerebellum by 40 dpo when PCs start expressing *Dab1*, and a clear cerebellar lamination featuring both a compact IGL and thick ML is established at this stage (Figure 8C). With respect to lizards, responding PCs in snakes are thus only exposed to one source of *Reln* produced by IGL GCs (Figure 8C). The asymmetry in cortical maturation at the onset of DAB1 activation, exposing PC to remarkably divergent molecular and spatial environment, might thus be responsible for alternative PC spatial layouts in our models. This hypothesis is coherent with the intact mature glial fiber organization and arrangement of PCs in radially oriented columns observed in the cerebellum of *B. fuliginosus* (Figures 8G,H), suggesting the presence of a proper guidance system supporting PC migration. Furthermore, previous observations on the asymmetric, fluctuating concentration of RELN during post-mitotic GCP migration in mice (Miyata et al., 1996) further suggest that RELN signaling emanating from GCPs is likely a major player in the positioning of PCs. Finally, the key importance of temporal and spatial patterning of GCs and PCs during cerebellar development have been demonstrated using transplantation experiments in rodents (Sotelo and Alvarado-Mallart, 1986, 1987a,b; Carletti et al., 2008), which showed that embryonic PCs transplanted in embryos at different developmental stages trigger variable ratios of PC misplacement in recipient ML, a phenotype related to host cortical maturation progression and EGL reduction (Carletti et al., 2008).

## Discussion

**FIGURE 9:**
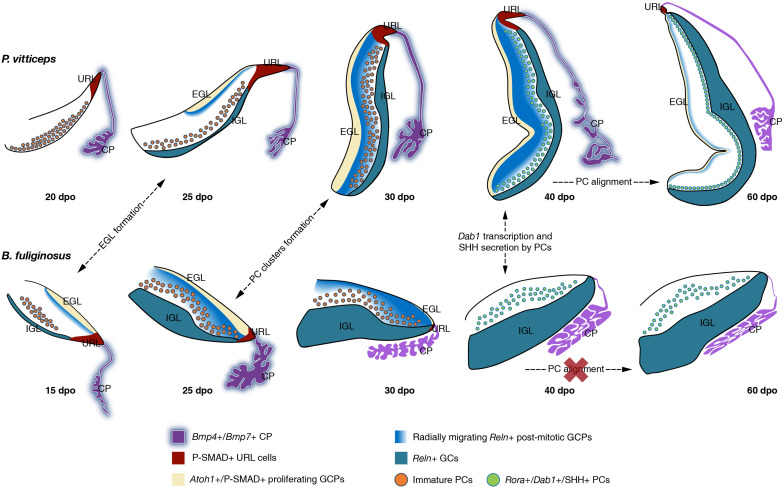
Overview of cerebellar development in squamate models. Schematic representation of the main molecular and cellular events at the origin of the divergent cortical lamination in *P. vitticeps* **(top row)** and *B. fuliginosus* **(bottom row)** cerebellum. The presence or absence (red cross) of major developmental processes at indicated embryonic stages are shown by dashed arrows. Key gene expression patterns in cerebellar regions and/or cell types identified in this study are indicated by different colors (see color code and symbols in bottom row). EGL, external granule layer; IGL, internal granule layer; URL, upper rhombic lip; CP, choroid plexus; GCP, granule cell progenitor; GC, granule cell; PC, Purkinje cell.

The generation, migration, and maturation of major cerebellar cell types are complex phenomena that impact whole-cerebellum morphogenesis, integrity, and function. Despite the overall conserved progenitor domains and salient physiological and morphological features of cerebellar GCs and PCs across vertebrates (Altman and Bayer, 1997), modifications in their developmental programs have been linked to the remarkable degree of cerebellar complexity achieved during vertebrate radiation in terms of both magnitude and spatial arrangement of neurons and foliation pattern. In this perspective, the absence of a typical EGL—defined as a distinct progenitor population covering the cerebellar pial surface and expressing *Atoh1*—in chondrichthyans and teleosts, but also the presence of a distinct, non-proliferative EGL in amphibians, indicate that the SHH-induced transit-amplifying phase in GCPs constitutes a hallmark of birds and mammals (Rodríguez-Moldes et al., 2008; Chaplin et al., 2010; Butts et al., 2014a; Pose-Méndez et al., 2016; Iulianella et al., 2019), likely allowing a massive GC production in a restricted developmental time window (Chaplin et al., 2010; Iulianella et al., 2019). In contrast, owing to the undetermined growth that characterizes the brain of anamniotes, some bony fishes and most likely chondrichthyans achieve extremely folded structures through continuous addition of GCs from stem progenitor pools throughout life (Candal et al., 2005; Zupanc et al., 2005; Rodríguez-Moldes et al., 2008; Chaplin et al., 2010; Kaslin et al., 2013; Butts et al., 2014b). Here, we show that squamate reptiles provide a new model system to examine both the role and evolutionary importance of EGL structure, neuronal arrangement patterns, but also signaling pathways in cerebellar morphogenesis (Figure 9). First, our data suggest that both the formation of a proliferative EGL and the expression of SHH by underlying PCs contribute to squamate cerebellogenesis, indicating that these processes are key developmental features of the amniote cerebellum. Furthermore, the direct comparison of our lizard and snake models strongly suggests the critical importance of spatiotemporal neuronal patterning and interaction between GCPs and PCs in defining cortical organization within amniotes. Indeed, the observed heterochronic shifts in URL activity and EGL maintenance in snakes is strongly expected to affect the dynamics of molecular interaction between these two cell types in snakes. Finally, although further experimental demonstrations would be needed, our data suggest the influence of key signaling pathways such as RELN on the behavior and spatial positioning of PCs in vertebrate cerebella. Altogether, our study provides new insights into the developmental origins of diversity in cerebellar cortical architecture and foliation pattern found in amniotes, including the remarkable array of PC spatial layouts identified in squamate reptiles, thus helping to complete our evolutionary understanding of vertebrate phenotypical diversification.

### Temporal Coupling Between EGL Formation and SHH Expression by Underlying PCs Regulates Squamate Cerebellar Complexity

The acute proliferation patterns of *Atoh1*-positive GCPs observed on the pial surface of the developing lizard and snake cerebellum unequivocally indicate the existence of a typical EGL in squamates, as previously identified in birds and mammals (Figure 9). In contrast, such secondary germinative zone has not been observed in a multitude of anamniote species (Rodríguez-Moldes et al., 2008; Chaplin et al., 2010; Butts et al., 2014b), including in metamorphic frogs where a subpial layer of cell migrating from the URL, resembling a non-proliferative EGL, has been described (Gona, 1972; Butts et al., 2014a). Our results, thus, document for the first-time that the EGL is a true amniote evolutionary novelty and not a distinctive feature of avian and mammalian cerebellogenesis. Importantly, however, we show that the EGL temporal dynamics differs considerably in our squamate models, the snake showing a precocious EGL decline and termination soon after its initial expansion, paralleling BMP expression pattern and activity in the adjacent CP and URL (Figure 9). In addition to differences in the timing of initial EGL formation, the heterogeneity in EGL duration between *P. vitticeps* and *B. fuliginosus* is expected to reside in the level of SHH signaling emanating from underlying PCs. Elegant experiments employing conditional mouse mutants have shown that the extent and complexity of cerebellar foliation is proportional to the intensity of SHH signaling (Corrales, 2006). A complete signal abrogation produces a precocious termination of GCP proliferation in the EGL, leading to the absence of foliation in the mutant cerebella, while more elaborated foliation patterns, resulting from EGL duration lengthening, parallel increase in SHH pathway activity. In a similar manner, the cerebella of our squamate models can be interpreted as phenotypic variants resulting from SHH signaling intensity modulation. Indeed, the complete decoupling between SHH pathway activation and EGL formation in the developing snake cerebellum recapitulates the situation occurring in mice totally depleted of SHH signaling after cerebellar primordium initiation. In such mutants, cerebellar foliation is inhibited because of a rapid depletion of GCPs from the EGL, and cerebella exhibit a dramatic reduction along the anteroposterior axis and lack any fissure. Likewise, the snake cerebellum is smooth and displays a significantly shorter pial surface extension (corresponding to mouse anteroposterior axis), when compared to the lizard counterpart that exhibits a more elaborated morphology. Furthermore, while both lizard and snake cerebella undergo an initial growth phase characterized by the lengthening and thickening of their cerebellar primordium, a second transit-amplifying phase of GCPs, featuring the formation of an incomplete and shallow fissure on the medial pial surface, occurs only in the lizard and is concurrent with SHH pathway activation and maintenance of proliferative EGL until postnatal stages. Although one or two transverse fissures dividing the cerebellum into different lobes have been previously reported in crocodilians and in some lizard species (Larsell, 1926, 1932), it is however unclear if the fissure observed in *P. vitticeps* is equivalent to the fissura prima, the first fissure to appear in the mammalian cerebellum, or simply linked to the inverted tilting of the lizard cerebellum. Moreover, the absence of foliation pattern in *P. vitticeps* despite the prolonged EGL maintenance suggests an overall low level of SHH signaling activity originating from lizard PCs (Corrales, 2006), which might be directly associated with the relatively reduced number of cerebellar PCs in squamates when compared to mammals (Wetts and Herrup, 1982; Bakalian et al., 1991, 1995; Frederic et al., 1992; Zanjani et al., 1992, 2004; and personal observations). Nevertheless, the persistence of EGL in lizards is also coherent with the formation of a more compact IGL as well as with the overall increase in cerebellum size, when compared to snakes.

Together, our findings provide new insights into the mechanisms determining cerebellar complexity in amniote lineages, including within squamate reptiles, thus suggesting an expanded role of SHH signaling in orchestrating cerebellum morphological complexity toward a broader evolutionary scope. In this perspective, it will be of great interest to functionally validate *in ovo* the hypothesis of a SHH-mediated transition between the unfoliated morphologies exhibited by squamates and the sophisticated architectures of birds and mammals once overcome the technical limitations posed by the use of non-canonical models. Furthermore, the maintenance of SHH signaling by *B. fuliginosus* PCs at both embryonic and postnatal stages could be an excellent experimental framework to assess the additional functions of this protein. In addition to cerebellar GCP proliferation enhancement, SHH has been shown to exert a fundamental role in expanding and maintaining an heterogeneous progenitor pool in the developing prospective cerebellar white matter in mice (Fleming et al., 2013). Moreover, its detection in adult mouse and rat PCs (Traiffort et al., 2002; Corrales, 2004; Lewis et al., 2004; Okuda et al., 2016), and its capability to regulate glutamate and ATP secretion from adult cerebellar astrocytes *in vitro* (Okuda et al., 2016), suggest the key importance of this protein in the adult cerebellum, likely in synaptic plasticity modulation.

### Importance of *Reln* Distribution Pattern in Cortical Maturation

A number of descriptive works on the cerebellar cortex of squamate species have emphasized the remarkable variation in PC spatial layouts, ranging from the almost regular monolayer exhibited by lizards to the scattered pattern featured by snakes (Larsell, 1926; ten Donkelaar and Bangma, 1992; Aspden et al., 2015; Wylie et al., 2017; Hoops et al., 2018). However, our recent quantitative work assessing PC topological distribution in a large squamate dataset depicted a much more complex scenario (Macrì et al., 2019). We revealed that a wide spectrum of PC spatial layouts is present both in snakes and lizards and parallels locomotor specialization, independently of phylogenetic relationships, likely reflecting different kinds of cerebellar mediated coordination. Our new data presented here unveil the developmental basis underlying such a wide variety of arrangements. We show that molecular markers expressed during early generation and maturation of PCs are conserved in both our models and coincide with the general PC developmental program described in other amniotes. No substantial difference is also evident between snakes and lizards during the initial radial migratory phase of post-mitotic PCPs, a process that, like in mice (Yuasa et al., 1993), is anticipated to occur in a RELN-independent fashion, as evidenced here by the lack of *Dab1*-positive PCs at early cerebellogenesis (Figure 9). However, despite the similar expression profile of both GCPs and PCs in our models, our findings outline that the dynamics of molecular interaction between these two cell types is altered in snakes. Especially, the rapid decline of radially migrating post-mitotic GCPs expressing *Reln*, a phenotype coherent with the observed heterochronic shifts in the timing and/or duration of URL activity and EGL maintenance, is expected to hamper the spatial reorganization of PCs at the end of their radial migratory phase in snakes (Figure 9). Although the exact mechanisms regulating the dispersal of PCs from PCC stage to monolayer organization are still unclear in mouse models (Rahimi-Balaei et al., 2018), our data corroborate previous studies proposing that post-mitotic GCPs are a major player in the positioning of PCs (Miyata et al., 1997; Jensen et al., 2002). More specifically, comparisons of our two models upon *Dab1* expression and activation indicate that either post-mitotic GCPs delaminating from the EGL and migrating across the PCCs or a combination of both EGL GCPs and IGL GCs are required for PC spatial reorganization in *P. vitticeps* (40 dpo; Figure 9). Coherent with these hypotheses, previous *in vitro* studies corroborated by *in vivo* observations have suggested that RELN is highly concentrated on the somata of GCPs as they move away from the EGL and contact the underlying PCCs, while the extracellular localization of the glycoprotein decreases and becomes only restricted to parallel fibers once GCs settle in the IGL (Miyata et al., 1996). Such switch in RELN concentration and localization might be associated to the transition from its positional instructive function during early cerebellogenesis to a role in PC position consolidation and dendritic maturation at later stages (Miyata et al., 1996). In such context, as the IGL GCs are the only source of *Reln* at the time of *Dab1* activation in snakes (40 dpo; Figure 9), the ML is likely permeated by a low concentration of RELN signals which accumulates on PC dendrites rather than somata, triggering only local responses that may favor synaptic plasticity but not cell body relocation. An additional obstacle to PCC dispersion in snakes could derive from a non-permissive spatial micro-environment. At 40 dpo, in fact, while lizard cortical segregation is only barely outlined, a clear lamination is already established in *B. fuliginosus* cerebellum, featuring a thick ML densely populated by GC parallel fibers that might constitute a physical barrier to PC repositioning.

Altogether, our observations further suggest a major role of the RELN pathway in controlling PC positioning in vertebrates (Caviness and Rakic, 1978; D’Arcangelo et al., 1995; Miyata et al., 1997; Jensen et al., 2002). However, despite the similarities in ectopic arrangement of PCs noticed between the adult cerebellum of snakes (this study and Macrì et al., 2019) and vertebrate mutants affecting the RELN pathway (Heckroth et al., 1989; D’Arcangelo et al., 1995; Sheldon et al., 1997; Ware et al., 1997; Gallagher et al., 1998; Trommsdorff et al., 1999; Nimura et al., 2019), significant phenotypic differences in cortical organization are noticeable. Especially, snake PCs are not found amassed in clusters or trapped in the IGL, as shown in mouse and zebrafish mutants with movement or behavioral defects (Inoue et al., 1990; Nimura et al., 2019). Instead, our developmental data in *B. fuliginosus* indicate that PCs are arranged in radially oriented columns, each consisting of a different number of cells protruding in the ML, likely reflecting the presence of a proper guidance system provided by an organized radial glia scaffold supporting their migration. This also contrasts with the aberrant and distorted organization of cerebellar radial glia, along with other types of glia located in different brain areas, observed in *reeler* mice (Bignami and Dahl, 1974; Terashima et al., 1985; Yuasa et al., 1991; Yuasa, 1996; Hartfuss et al., 2003; Weiss et al., 2003) and *Reln* knockout zebrafish (Nimura et al., 2019). These differences suggest that *Reln* expression initiating at early stages of cerebellogenesis in differentiating GCPs, a distribution pattern similar among squamate models (Figure 9) but also in mice (Miyata et al., 1996; Rice et al., 1998), could be important for the maturation and migration of other cerebellar cell types, most likely developing glia.

In conclusion, our characterization of squamate cerebellogenesis indicates a complex and dynamic crosstalk between developing cerebellar neurons in a new major group of vertebrates, and strongly suggests the existence of variations from a common amniote developmental blueprint for cerebellar histogenesis. Furthermore, our comparative gene expression analysis, conducted on species featuring extremely divergent cortical arrangements, supports a pivotal role of heterochrony in generating phenotypic variation, but also give new insights into the morphogenetic and molecular underpinnings at the base of squamate cerebellar cortex diversification. Altogether, these findings provide a new perspective on the complex evolutionary processes and morphogenetic dynamics underlying increased complexity of the amniote cerebellum, highlighting the potential offered by squamate models to expand our understanding of such unique neurodevelopmental landscape.

## Materials and Methods

### Sample Collection

*P. vitticeps* and *B. fuliginosus* embryonic series as well as hatchlings and juveniles (2–6 months after hatching) were obtained from our animal facility at the University of Helsinki. Fertilized eggs were incubated on a moistened vermiculite substrate at 29.5°C, as previously described (Ollonen et al., 2018). Embryos were collected at regular intervals spanning the entire post-ovipositional period (about 60 days in both species), and embryonic staging was performed on the basis of external morphology according to developmental tables available for the two species (Boback et al., 2012; Ollonen et al., 2018). A minimum of three biological replicates were analyzed for each developmental time point in the different experiments.

### Micro-CT Scan and 3D Brain Reconstructions

High-resolution 3D CT-scans of *P. vitticeps* and *B. fuliginosus* heads were performed at the University of Helsinki imaging facility using Skyscan 1272 (Brucker, Belgium). Prior to micro-CT scanning, to allow reptile brain tissue visualization, samples were treated with 1% iodine solution as previously described (Metscher, 2009; Macrì et al., 2019). The following scan parameters were used: filter: Al 0.25 mm; source voltage: 60 kV; source current: 166 μA; voxel size: 12 μm; rotation steps: 0,2°; frame averaging: 8. Scans were reconstructed using NRecon 1.7.0.4 software (Bruker) and 3D volume rendering as well as segmentation were performed using the software Amira 5.5.0 (Thermo Fisher Scientific, United States). Both the whole-brain and isolated cerebellum were segmented, thus allowing assessment of volumetric measurements of these structures. The accuracy of all generated 3D models was carefully controlled along the three anatomical planes, as described (Macrì et al., 2019).

### Nissl Staining and Immunohistochemistry

Embryonic heads were fixed overnight in 4% paraformaldehyde (PFA) at 4°C and dehydrated through a series of washes in PBS containing increasing methanol concentrations (25, 50, 75, and 100%). For optimal sectioning, the brains of both juvenile individuals and embryos presenting an advanced degree of skull ossification were dissected from the braincase before fixation. After dehydration, samples were paraffin-embedded and microtome-sectioned at 9 μm. For Nissl staining, sections were deparaffinized in xylene, rehydrated in decreasing ethanol concentration solutions (100, 95, 70%), and rinsed first in running tap water and successively in distilled water. Staining was performed in 0.1% (w/v) cresyl violet solution (cresyl violet acetate, Sigma-Aldrich, Cat# C5042) in distilled water, at 37°C. Once stained, sections were rinsed in distilled water and immersed in differentiating solution (95% ethanol in distilled water) for 10 min, dehydrated in 100% ethanol and cleared in xylene before mounting slides with DPX medium (Sigma-Aldrich, Cat# 06522). IHC on sections was conducted as previously described (Salomies et al., 2019), utilizing the following primary antibodies: PCNA (1:500, mouse monoclonal, BioLegend, Cat# 307901, RRID: AB_314691), CALB1 (1:300, rabbit polyclonal, Swant, Cat# CB38, RRID: AB_10000340), phospho histone H3 Ser10 (PH3, 1:500, rabbit polyclonal, Abcam, Cat# ab5176, RRID: AB_304763), SHH (1:400, rabbit polyclonal, LifeSpan Cat# LS-C40460, RRID:AB_2285962), LHX1 (1:400, rabbit polyclonal, LifeSpan, Cat# LS-C16214, RRID: AB_2135639), ZIC1/2/3 (1:300, rabbit polyclonal, LifeSpan, Cat# LS-C118695), phospho SMAD1/5/9 (P-SMAD1/5/9; 1:500, rabbit polyclonal, Cell Signaling Technology, Cat# 13820, RRID: AB_2493181), P-DAB1 (1:400, rabbit polyclonal, Biorbyt, Cat# orb156526), and glial fibrillary acidic protein (GFAP, 1:200, mouse monoclonal, Lifespan, Cat # LS-C357895). Alexa Fluor−488 (1:500-1:1000, goat anti−mouse IgG, Thermo Fisher Scientific, Cat# A-11001, RRID: AB_2534069) and Alexa Fluor−568 (1:500-1:1000, goat anti−rabbit IgG, Thermo Fisher Scientific, Cat# A−11011, RRID: AB_143157) were used as secondary antibodies. Slide mounting and nuclear counterstaining were carried out with Fluoroshield mounting medium (Sigma−Aldrich) containing 4′, 6′−diamidino−2−phenylindole (DAPI).

### *In situ* Hybridization

*In situ* hybridization on paraffin sections was performed as previously described (Eymann et al., 2019), using digoxigenin (DIG)-labeled antisense riboprobes corresponding to atonal bHLH transcription factor 1 (*Atoh1*), retinoic acid receptor-related orphan receptor alpha (*Rora*), very-low-density-lipoprotein receptor (*vldlr*), Reelin (*Reln*), disabled-1 (*Dab1*), bone morphogenetic protein 4 (*Bmp4*), and bone morphogenetic protein 7 (*Bmp7*). Riboprobes were generated based on publicly available cDNA and/or genome sequences available for lizards and snakes, including *P. vitticeps* (Georges et al., 2015) and *Pantherophis guttatus* (*P. guttatus*; Ullate-Agote et al., 2014), as well as on newly produced nucleotide sequences for *B. fuliginosus* (Table 1). *In situ* hybridization was performed on paraffin sections as described previously (Eymann et al., 2019), using a hybridization temperature of 65°C. Following overnight hybridization, sections were washed and incubated with alkaline phosphatase-conjugated anti-DIG antibodies (1:2500, sheep polyclonal, Sigma-Aldrich, cat# 11093274910, RRID: AB_2734716). For colorimetric visualization of hybridization, sections were stained with a solution containing 5-bromo-4-chloro-3-indolyl phosphate and nitro blue tetrazolium.

**TABLE 1:** Details on nucleotide sequences used to obtain riboprobes in ISH experiments.

Gene name	Species	Sequence length (bp)	Sequence accession number (NCBI)	Nucleotide sequence position
*Bmp4*	*P. vitticeps*	670	XM_020813026	1…670
*Bmp7*	*P. vitticeps*	584	XM_020797472	50…633
*Atoh1*	*P. vitticeps*	966	CEMB01011076	706854…707819
*Rora*	*P. vitticeps*	942	XM_020784749	184…1125
*Vldlr*	*P. vitticeps*	830	XM_020801543	4592…5421
*Reln*	*P. vitticeps*	1237	XM_020790001	7804…9040
*Dab1*	*P. vitticeps*	848	XM_020789863	1064…1911
*Bmp4*	*P. guttatus*	785	XM_034433399	646…1430
*Bmp7*	*P. guttatus*	784	XM_034433071	502…1285
*Atoh1*	*B. fuliginosus*	623	MT993473	1…623
*Rora*	*B. fuliginosus*	907	MT993472	1…907
*Reln*	*B. fuliginosus*	927	MT993475	1…927
*Dab1*	*B. fuliginosus*	612	MT993474	1…612

### Image Acquisition and Processing

Histological preparations as well as IHC and ISH slides were imaged using a Nikon Eclipse 90i microscope. Nikon DS−Fi U3 and Hamamatsu Flash4.0 cameras were used for capturing bright field and fluorescence images, respectively. Images larger than the microscope field of view were acquired as partially overlapping tiles and successively stitched together in Adobe Photoshop CC (RRID:SCR_014199) using the photomerge function. To improve visualization, linear levels were adjusted in Adobe Photoshop CC.

## Data Availability Statement

The datasets presented in this study can be found in online repositories. The names of the repository/repositories and accession number(s) can be found below: https://www.ncbi.nlm.nih.gov/genbank/, MT993472; https://www. ncbi.nlm.nih.gov/genbank/, MT993473; https://www.ncbi.nlm.nih.gov/genbank/, MT993474; https://www.ncbi.nlm.nih.gov/genbank/.

## Ethics Statement

The animal study was reviewed and approved by Laboratory Animal Centre (LAC) of the University of Helsinki and/or National Animal Experiment Board (ELLA) in Finland (license numbers ESLH-2007-07445/ym-23, ESAVI/7484/04.10.07/2016, and ESAVI/13139/04.10.05/2017).

## Author Contributions

SM performed all the experiments. All authors designed the experimental approach, collected and prepared the samples, analyzed the data, wrote the manuscript, and read and approved the final manuscript.

## Conflict of Interest

The authors declare that the research was conducted in the absence of any commercial or financial relationships that could be construed as a potential conflict of interest.

## Supplementary Material

Supplementary Figure 1 Molecular characterization of postnatal squamate cerebellum. **(A,B)** Double IHC for PCNA (green staining) and SHH (red) markers at 15 days post-hatching (dph) **(A)** or ISH for *Rora* at juvenile stage **(B)** in the cerebellum of *P. vitticeps*. The arrowhead in panel **(A)** indicates the position of the incomplete fissure on the cerebellar pial surface. Insets in **(A)** show high magnifications of proliferating GCPs (green) or SHH-positive PCs (red). **(C,D)** ISH for *Rora* **(C)** or *Reln* **(D)** in the cerebellum of juvenile *B. fuliginosus*. PS, pial surface; IGL, internal granule layer; VS, ventricular surface. Scale bars: 50 μm.
