# Supplementary material for: Heterochronic Developmental Shifts Underlying Squamate Cerebellar Diversity Unveil the Key Features of Amniote Cerebellogenesis
Source: Front Cell Dev Biol. 2020 Oct 22;8:593377. doi: 10.3389/fcell.2020.593377 (PMC7642464; doi:10.3389/fcell.2020.593377)
Supplement: Supplementary Figure 1 — Molecular characterization of postnatal squamate cerebellum. (A,B) Double IHC for PCNA (green staining) and SHH (red) markers at 15 days post-hatching (dph) (A) or ISH for Rora at juvenile stage (B) in the cerebellum of P. vitticeps. The arrowhead in panel (A) indicates the position of the incomplete fissure on the cerebellar pial surface. Insets in (A) show high magnifications of proliferating GCPs (green) or SHH-positive PCs (red). (C,D) ISH for Rora (C) or Reln (D) in the cerebellum of juvenile B. fuliginosus. PS, pial surface; IGL, internal granule layer; VS, ventricular surface. Scale bars: 50 μm. [file Data_Sheet_1.PDF]

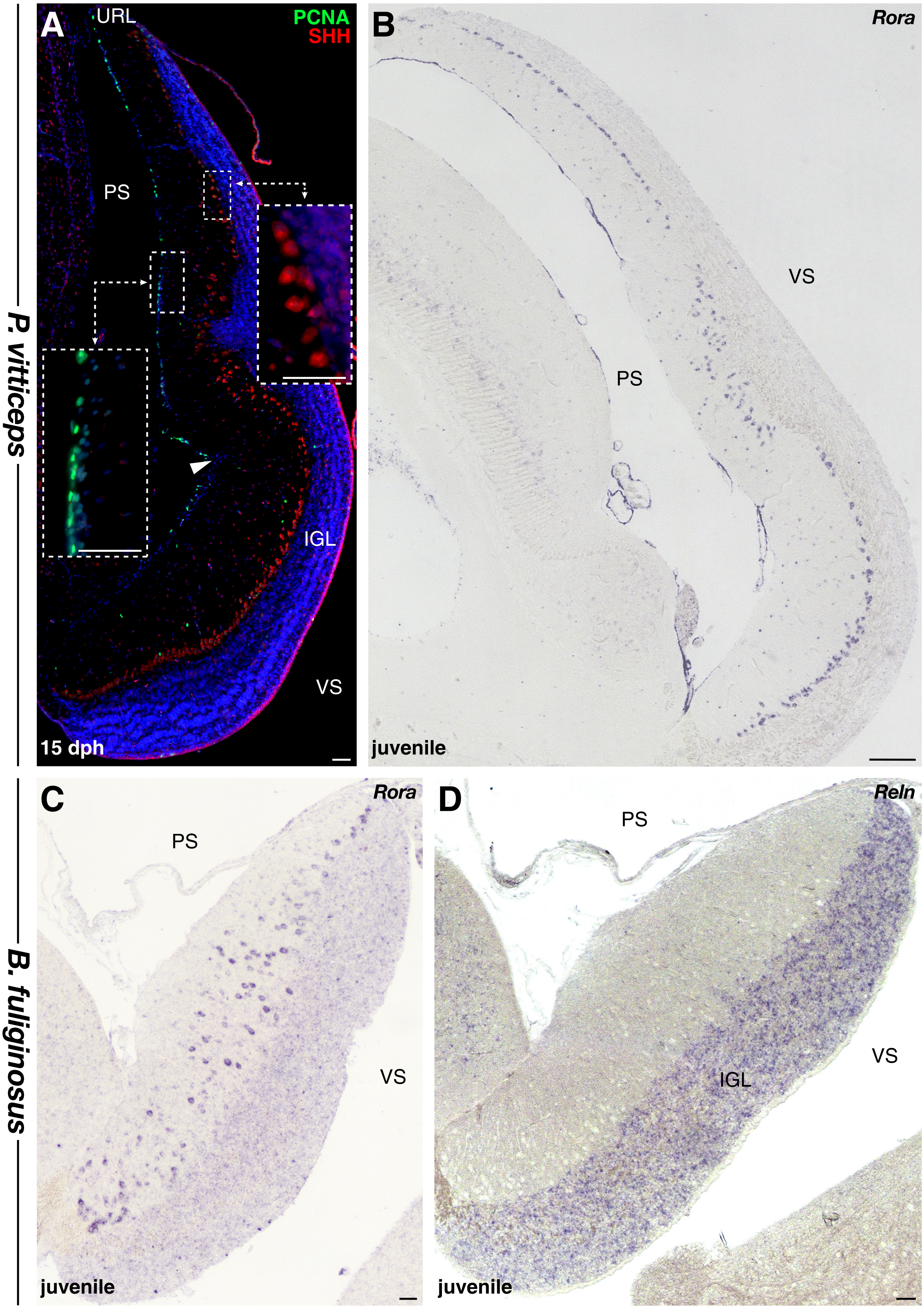

Fig. S1. Molecular characterization of postnatal squamate cerebellum. (A,B) Double IHC for PCNA (green staining) and SHH (red) markers at 15 days post-hatching (dph; A) or ISH for *Rora* at juvenile stage (B) in the cerebellum of *P. vitticeps*. The arrowhead in (A) indicates the position of the incomplete fissure on the cerebellar pial surface. Insets in (A) show high magnifications of proliferating GCPs (green) or SHH-positive PCs (red). (C,D) ISH for *Rora* (C) or *Reln* (D) in the cerebellum of juvenile *B. fuliginosus*. Abbreviations: PS, pial surface; IGL, internal granule layer; VS, ventricular surface. Scale bars: 50 µm.
